# Supplementary material for: Enzymatic hydrolysis of starch from the anthocyanin extraction residue (AER-starch) with ultrasound pretreatment: A techno-economic assessment
Source: PLoS One. 2026 Mar 2;21(3):e0343968. doi: 10.1371/journal.pone.0343968 (PMC12952594; doi:10.1371/journal.pone.0343968)
Supplement: S1 Table — This Table summarizes the parameters used in the techno-economic analysis. (DOCX) [file pone.0343968.s001.docx]

S1 Table. Economic analysis assumptions for the enzymatic hydrolysis process.

| Parameter | Value/description |
| --- | --- |
| Project lifetime (years) | 15 |
| Discount rate (%) | 7 |
| Income taxes (%) | 25 |
| Depreciation method | Straight-line |
| Depreciation period (years) | 10 |
| Currency | US Dollar (USD) |
| Reference year | 2025 (all costs expressed in constant 2025 USD) |
| Inflation index | 2025 (the capital cost was adjusted using SuperPro V14 built-in values of the Chemical Engineering Cost Index) |
| Plant availability (days/year) | 330 |
| Working capital | Estimated to cover 30 days of expenses for labor, raw materials, utilities and waste treatment |
| Labor cost | Based on the Colombian minimum wage regulations, as established by the national government. This information can be consulted on the official website of the Colombian government: <https://www.funcionpublica.gov.co/eva/gestornormativo/norma.php?i=257156> |
| Resin replacement basis | Load an ion exchange column, the empty bed contact time was 3.00 min, the binging capacity of the resin was 80 g/L (ion mass), the breakthrough time was 2.00 h.  Load a GAC column, the empty bed contact time was 1.75 min, the mass of adsorbing components was 98 mg per g of adsorbent, the breakthrough time was 2.00 h. |
| Evaporation energy (Total thermal energy demand) | 4539 kW-h per batch |
