# Supplementary material for: Enzymatic hydrolysis of starch from the anthocyanin extraction residue (AER-starch) with ultrasound pretreatment: A techno-economic assessment
Source: PLoS One. 2026 Mar 2;21(3):e0343968. doi: 10.1371/journal.pone.0343968 (PMC12952594; doi:10.1371/journal.pone.0343968)
Supplement: S2 Table — This Table summarizes the major material flows and utility demands obtained from the SuperPro Designer v14® simulation. (DOCX) [file pone.0343968.s002.docx]

S2 Table. Summarized mass and energy balances for the hydrolysis process.

|  | Inlet stream | Mass composition (%) | | Outlet stream | Mass composition (%) | | Key performance parameters | Energy flow in (kW h^-1^) | Energy flow out (kg h^-1^) |
| --- | --- | --- | --- | --- | --- | --- | --- | --- | --- |
| Starch hydrolysis (R-101) | S-109  5000 kg batch^-1^ | Alpha amylase | 0.0166 | S-103  5001 kg batch^-1^ | Alpha amylase | 0.0166 | Reaction molar stoichiometry: 1 AER-starch + 9 Water → 10 DPn* | 1868.93 | 2006.51 |
|  |  | Ash | 0.0167 |  | Ash | 0.0166 |  |  |  |
|  |  | CaCl2 | 0.0931 |  | CaCl2 | 0.0931 |  |  |  |
|  |  | Soluble protein | 0.082 |  | Soluble protein | 0.082 |  |  |  |
|  |  | Solubles | 0.0412 |  | Solubles | 0.0412 |  |  |  |
|  |  | AER-starch | 29.6882 |  | DPn^*^ | 30.2267 |  |  |  |
|  |  | Sulfur dioxide | 0.0186 |  | Sulfur dioxide | 0.0186 |  |  |  |
|  |  | Water | 70.0436 |  | Water | 69.5052 |  |  |  |
| Saccharification (R-102) | S-111  5002 kg batch^-1^ | Alpha amylase | 0.0166 | S-105  5001 kg batch^-1^ | Alpha amylase | 0.0166 | Reaction molar stoichiometry:8 DPn + 148 Water → 153 Glucose + 2 Maltose + 1 Maltotriose | 1365.93 | 1365.93 |
|  |  | Ash | 0.0166 |  | Ash | 0.0166 |  |  |  |
|  |  | CaCl2 | 0.0931 |  | CaCl2 | 0.0931 |  |  |  |
|  |  | DPn | 30.2267 |  | DPn | 0.0906 |  |  |  |
|  |  | Amyloglucosidase | 0.0181 |  | Amyloglucosidase | 0.0181 |  |  |  |
|  |  | HCl | 0.02 |  | Glucose | 31.8163 |  |  |  |
|  |  | Soluble protein | 0.082 |  | HCl | 0.02 |  |  |  |
|  |  | Solubles | 0.0412 |  | Maltose | 0.7919 |  |  |  |
|  |  | Sulfur dioxide | 0.0186 |  | Maltotriose | 0.5812 |  |  |  |
|  |  | Water | 69.4671 |  | Soluble protein | 0.082 |  |  |  |
|  |  |  |  |  | Solubles | 0.0412 |  |  |  |
|  |  |  |  |  | Sulfur dioxide | 0.0121 |  |  |  |
|  |  |  |  |  | Water | 66.4203 |  |  |  |
| Rotary vacuum filtration (RVF-101) | S-105  5001 kg batch^-1^ | Alpha amylase | 0.0166 | S-112  5001 kg batch^-1^ | Ash | 0.0166 | Enzymes removed: 100% | 454.97 | 454.97 |
|  |  | Ash | 0.0166 |  | CaCl2 | 0.0931 |  |  |  |
|  |  | CaCl2 | 0.0931 |  | DPn | 0.0906 |  |  |  |
|  |  | DPn | 0.0906 |  | Glucose | 31.8163 |  |  |  |
|  |  | Amyloglucosidase | 0.0181 |  | HCl | 0.02 |  |  |  |
|  |  | Glucose | 31.8163 |  | Maltose | 0.7919 |  |  |  |
|  |  | HCl | 0.02 |  | Maltotriose | 0.5812 |  |  |  |
|  |  | Maltose | 0.7919 |  | Soluble protein | 0.082 |  |  |  |
|  |  | Maltotriose | 0.5812 |  | Solubles | 0.0412 |  |  |  |
|  |  | Soluble protein | 0.082 |  | Sulfur dioxide | 0.0121 |  |  |  |
|  |  | Solubles | 0.0412 |  | Water | 66.455 |  |  |  |
|  |  | Sulfur dioxide | 0.0121 |  |  |  |  |  |  |
|  |  | Water | 66.4203 |  |  |  |  |  |  |
| Ion Exchange (INX-101) | S-113  5001 kg batch^-1^ | Ash | 0.0166 | S-114  5106 kg batch^-1^ | DPn | 0.0866 | Component binding (100%): ash, CaCl2, HCl, Sulfur dioxide. Component binding (20%): soluble protein, solubles. Component binding (0,1%): DPn, glucose, maltose, maltrotriose. | 620.26 | 620.26 |
|  |  | CaCl2 | 0.0931 |  | Glucose | 30.4037 |  |  |  |
|  |  | DPn | 0.0906 |  | Maltose | 0.7568 |  |  |  |
|  |  | Glucose | 31.8163 |  | Maltotriose | 0.5554 |  |  |  |
|  |  | HCl | 0.1449 |  | Soluble protein | 0.0381 |  |  |  |
|  |  | Maltose | 0.7919 |  | Solubles | 0.0192 |  |  |  |
|  |  | Maltotriose | 0.5812 |  | Water | 68.1402 |  |  |  |
|  |  | Soluble protein | 0.082 | Waste of INX-101  696 kg batch^-1^ | Ash | 0.1298 |  |  |  |
|  |  | Solubles | 0.0412 |  | CaCl2 | 0.7268 |  |  |  |
|  |  | Sulfur dioxide | 0.0121 |  | DPn | 0.0007 |  |  |  |
|  |  | Water | 66.3301 |  | Glucose | 0.2455 |  |  |  |
|  | NaOH to INX-101  200 kg batch^-1^ | NaOH | 3.974 |  | HCl | 1.2242 |  |  |  |
|  |  | Water | 96.026 |  | Maltose | 0.0061 |  |  |  |
|  | HCL to INX-101  201 kg batch^-1^ | HCl | 3.7563 |  | Maltotriose | 0.0045 |  |  |  |
|  |  | Water | 96.2437 |  | NaOH | 0.1321 |  |  |  |
|  | Washing water  400 kg batch^-1^ | Water | 100 |  | Soluble protein | 0.0768 |  |  |  |
|  |  |  |  |  | Solubles | 0.0386 |  |  |  |
|  |  |  |  |  | Sulfur dioxide | 0.0965 |  |  |  |
|  |  |  |  |  | Water | 97.3184 |  |  |  |
| GAC Adsorption (GAC-101) | S-117  5106 kg batch^-1^ | DPn | 0.0866 | S-115  5101.41 kg batch^-1^ | DPn | 0.0866 | Component binding (90%): soluble protein, Solubles. Component binding (0,1%): DPn, glucose, maltose, maltrotriose. | 516.94 | 516.94 |
|  |  | Glucose | 30.4037 |  | Glucose | 30.4037 |  |  |  |
|  |  | Maltose | 0.7568 |  | Maltose | 0.7568 |  |  |  |
|  |  | Maltotriose | 0.5554 |  | Maltotriose | 0.5554 |  |  |  |
|  |  | Soluble protein | 0.0381 |  | Soluble protein | 0.0381 |  |  |  |
|  |  | Solubles | 0.0192 |  | Solubles | 0.0192 |  |  |  |
|  |  | Water | 68.1402 |  | Water | 68.1402 |  |  |  |
|  | NaOH to GAC-101  76.59 kg batch^-1^ | NaoH | 0.8083 | Waste of GAC-101  157.77 kg batch^-1^ | DPn | 0.0031 |  |  |  |
|  |  | Water | 99.1917 |  | Glucose | 1.1045 |  |  |  |
|  | Water to GAC-101  76.59 kg batch^-1^ | Water | 100 |  | Maltose | 0.0275 |  |  |  |
|  |  |  |  |  | Maltotriose | 0.0202 |  |  |  |
|  |  |  |  |  | NaOH | 0.3927 |  |  |  |
|  |  |  |  |  | Soluble protein | 1.246 |  |  |  |
|  |  |  |  |  | Solubles | 0.6223 |  |  |  |
|  |  |  |  |  | Water | 96.5837 |  |  |  |
| Evaporation (EV-101) | S-116  5101.41 kg batch^-1^ | DPn | 0.0866 | Glucose syrup  1581.41 kg batch^-1^ | DPn | 0.0866 | Final water mass fraction: 5% | 508.09 | 508.09 |
|  |  | Glucose | 30.4037 |  | Glucose | 93.1216 |  |  |  |
|  |  | Maltose | 0.7568 |  | Maltose | 1.1892 |  |  |  |
|  |  | Maltotriose | 0.5554 |  | Maltotriose | 1.2396 |  |  |  |
|  |  | Soluble protein | 0.0381 |  | Soluble protein | 0.0085 |  |  |  |
|  |  | Solubles | 0.0192 |  | Solubles | 0.0043 |  |  |  |
|  |  | Water | 68.1402 |  | Water | 4.3502 |  |  |  |

^*^DPn: Higher sugars
