# Supplementary material for: Enzymatic hydrolysis of starch from the anthocyanin extraction residue (AER-starch) with ultrasound pretreatment: A techno-economic assessment
Source: PLoS One. 2026 Mar 2;21(3):e0343968. doi: 10.1371/journal.pone.0343968 (PMC12952594; doi:10.1371/journal.pone.0343968)
Supplement: Attachment — Submitted filename: Response to Reviewers.docx [file pone.0343968.s004.docx]

Journal Requirements: The reply and manuscript’s text adjustments of Reviewer #1 comments were highlighted in green. We thank the academic editor and PLOS ONE staff thorough review of the manuscript.

1. Please ensure that your manuscript meets PLOS ONE's style requirements, including those for file naming. The PLOS ONE style templates can be found at

https://journals.plos.org/plosone/s/file?id=wjVg/PLOSOne_formatting_sample_main_body.pdf and

https://journals.plos.org/plosone/s/file?id=ba62/PLOSOne_formatting_sample_title_authors_affiliations.pdf

Reply: Thank you for your feedback. We have reviewed the style requirements.

2. Please note that PLOS One has specific guidelines on code sharing for submissions in which author-generated code underpins the findings in the manuscript. In these cases, we expect all author-generated code to be made available without restrictions upon publication of the work. Please review our guidelines at https://journals.plos.org/plosone/s/materials-and-software-sharing#loc-sharing-code and ensure that your code is shared in a way that follows best practice and facilitates reproducibility and reuse.

Reply: Thank you for your feedback. All relevant data are provided within the manuscript. The process simulations were performed using SuperPro Designer v14®, a commercially licensed software. No author-generated code was used in this study. As the software is proprietary, it cannot be shared. However, all model parameters, assumptions, and input data required to reproduce the simulations have been fully described and are available in the manuscript.

3. Thank you for stating the following financial disclosure:

“Institución Universitaria Colegio Mayor de Antioquía. Convocatoria de investigación. FCSA23.”

Please state what role the funders took in the study. If the funders had no role, please state: "The funders had no role in study design, data collection and analysis, decision to publish, or preparation of the manuscript."

If this statement is not correct you must amend it as needed.

Please include this amended Role of Funder statement in your cover letter; we will change the online submission form on your behalf.

Reply: Thank you for your feedback. We have reviewed it.

4. Thank you for stating the following in the Acknowledgments Section of your manuscript:

“The authors are grateful to COLMAYOR for financial support.”

We note that you have provided funding information that is not currently declared in your Funding Statement. However, funding information should not appear in the Acknowledgments section or other areas of your manuscript. We will only publish funding information present in the Funding Statement section of the online submission form.

Please remove any funding-related text from the manuscript and let us know how you would like to update your Funding Statement. Currently, your Funding Statement reads as follows:

“Institución Universitaria Colegio Mayor de Antioquía. Convocatoria de investigación. FCSA23.”

Please include your amended statements within your cover letter; we will change the online submission form on your behalf.

Reply: Thank you for your feedback. We have reviewed it.

5. We note that your Data Availability Statement is currently as follows: All relevant data are within the manuscript and its Supporting Information files.

Please confirm at this time whether or not your submission contains all raw data required to replicate the results of your study. Authors must share the “minimal data set” for their submission. PLOS defines the minimal data set to consist of the data required to replicate all study findings reported in the article, as well as related metadata and methods (https://journals.plos.org/plosone/s/data-availability#loc-minimal-data-set-definition).

For example, authors should submit the following data:

- The values behind the means, standard deviations and other measures reported;

- The values used to build graphs;

- The points extracted from images for analysis.

Authors do not need to submit their entire data set if only a portion of the data was used in the reported study.

If your submission does not contain these data, please either upload them as Supporting Information files or deposit them to a stable, public repository and provide us with the relevant URLs, DOIs, or accession numbers. For a list of recommended repositories, please see https://journals.plos.org/plosone/s/recommended-repositories.

If there are ethical or legal restrictions on sharing a de-identified data set, please explain them in detail (e.g., data contain potentially sensitive information, data are owned by a third-party organization, etc.) and who has imposed them (e.g., an ethics committee). Please also provide contact information for a data access committee, ethics committee, or other institutional body to which data requests may be sent. If data are owned by a third party, please indicate how others may request data access.

Reply: Thank you for your feedback. We confirm that all raw data required to replicate the results of our study have been made publicly available. The complete dataset is openly accessible on Zenodo at the following DOI:

<https://doi.org/10.5281/zenodo.18257051>

6. We note that you have included the phrase “data not shown” in your manuscript. Unfortunately, this does not meet our data sharing requirements. PLOS does not permit references to inaccessible data. We require that authors provide all relevant data within the paper, Supporting Information files, or in an acceptable, public repository. Please add a citation to support this phrase or upload the data that corresponds with these findings to a stable repository (such as Figshare or Dryad) and provide and URLs, DOIs, or accession numbers that may be used to access these data. Or, if the data are not a core part of the research being presented in your study, we ask that you remove the phrase that refers to these data.

Reply: Thank you for your comment. We have revised the manuscript accordingly. The phrase “data not shown” has been removed and all data have now been made publicly available. These data are included in the dataset deposited in Zenodo, accessible at the following DOI:

<https://doi.org/10.5281/zenodo.17872033>

7. If the reviewer comments include a recommendation to cite specific previously published works, please review and evaluate these publications to determine whether they are relevant and should be cited. There is no requirement to cite these works unless the editor has indicated otherwise.

Reply: Thank you for your feedback. We have reviewed it.

Reviewers' comments:

Reviewer #1: The reply and manuscript’s text adjustments of Reviewer #1 comments were highlighted in yellow. We thank the Reviewer for the thorough review of the manuscript.

1. The manuscript would benefit from a clearer mechanistic explanation of the ultrasound pretreatment, particularly at higher starch concentrations, to strengthen the scientific rationale presented in the Introduction section.

Reply: Thank you for your feedback. We have added the following paragraph to the discussion:

At higher starch concentrations, the solution viscosity increased, diminishing the performance of the ultrasound pretreatment. The increase in the starch solution viscosity limits the bubble growth and collapse, leading to a significant rise in the cavitation threshold [27]. Although higher amplitudes can partially compensate for the effect of high viscosity, amplitudes above 60% could not be used in this study to preserve probe integrity and ensure equipment maintenance. Consequently, ultrasound pretreatment becomes less effective at higher AER-starch concentrations.

2. A more detailed discussion of the observed combination of high SC and lower HD would enrich the understanding of potential oligosaccharide formation and saccharification completeness.

Reply: Thank you for your feedback. We have added the following paragraph to the discussion:

In general, shorter hydrolysis times resulted in higher SC values but lower HD values, suggesting the accumulation of partially hydrolyzed oligosaccharides. In this study, although longer saccharification times increased HD values, complete saccharification to glucose was not achieved, and the positive effects of the ultrasound pretreatment were limited. Under the conditions studied, starch accessibility was unlikely to have been the limiting factor, and the saccharification step determined hydrolysis completeness. Overall, ultrasound at higher AER-starch concentrations had a limited impact on HD and no effect on SC. Therefore, the ultrasound pretreatment does not improve the hydrolysis at high AER-starch concentrations.

3. Sections 2.2 & 2.3, Including thermal-only controls and confirming enzyme stability under SEH conditions would enhance the robustness of the experimental design and improve interpretation of ultrasound-specific effects.

Reply: Thank you for this observation. The SEH was explored as a strategy to reduce overall processing time by combining the liquefaction and saccharification steps. However, the hydrolysis yields obtained under SEH conditions were lower than those achieved in the sequential process. This behavior can be explained by the different optimal temperature requirements of the enzymes employed. The α-amylase used is an industrial enzyme designed to operate at high temperatures (90 °C), whereas amyloglucosidase exhibits optimal activity at lower temperatures (60 °C). Operating SEH at an intermediate temperature (75 °C) represented a compromise that likely reduced amyloglucosidase activity and limited saccharification efficiency as we mentioned in the text. These results highlight the importance of enzyme thermal compatibility when implementing SEH, rather than indicating intrinsic limitations of the process or enzyme performance.

4. Section 2.3, Reporting additional ultrasound parameters such as delivered power, acoustic energy density, and temperature control would support reproducibility and alignment with literature.

Reply: Thank you for your feedback. We have added the following information to the manuscript:

Ultrasound pretreatment was performed using a 750 W ultrasonic homogenizer (Cole-Parmer, Vernon Hills, IL, USA) equipped with temperature control (30°C). AER-starch mixtures were treated for 30 minutes at an amplitude of 60% in pulse mode (2 seconds ON, 2 seconds OFF), with an estimated delivered power of 600 W and an acoustic energy density of 592 W cm^-^².

5. Section 3.1, Providing a more detailed explanation for cases where SC exceeds 90% but HD remains lower would enrich understanding of oligosaccharide formation and saccharification completeness.

Reply: Thank you for your feedback. We have added the following information to the manuscript:

The combination of higher SC values (>90%) and lower HD values, observed mainly in the simultaneous process, can be explained by the formation and accumulation of soluble oligosaccharides rather than by complete saccharification to glucose. SC reflects the solubilization of AER-starch into soluble compounds, whereas HD (determined by the DNS method) quantifies reducing sugars such as glucose and short-chain saccharides. In CEH and UCEH, higher SC and SC values were obtained, indicating that starch solubilization followed by saccharification was an effective process. In contrast, lower values of SC and HD were obtained in SEH because the hydrolysis was performed at a temperature outside the optimal range recommended by the enzyme supplier. Although ultrasound pretreatment enhances starch solubilization, it did not significantly promote the enzymatic conversion of soluble compounds into glucose under simultaneous hydrolysis conditions. Thus, while disruption of starch granules and generation of soluble intermediate compounds are effective during pretreatment and liquefaction, saccharification completeness is governed by temperature and pH conditions rather than starch accessibility alone.

6. Sections 3.1 & 3.2, Clarifying the varying influence of ultrasound across experiments would help present a more cohesive interpretation, as SEH and CEH results differ in several places.

Reply: Thank you for your feedback. We agree that clarifying the differing influence of ultrasound across CEH and SEH is important for a cohesive interpretation of the results. Based on your suggestion and related comments from the other reviewer, we revised Sections 3.1 and 3.2 to explicitly distinguish the role of ultrasound under each hydrolysis strategy.

7. Including mass and energy balance summaries would support validation of the process model and clarify how each unit operation contributes to overall process performance.

Reply: Thank you for the comment. This information has been compiled and is now presented as supplementary material in S2 Table.

8. In Techno Economic Evaluation (Section 2.6), Providing justification for key assumptions such as labor cost, resin replacement rates, evaporation energy, and depreciation, would improve transparency of the economic modeling.

Reply: Thank you for the comment. This information has been compiled and is now presented as supplementary material in S1 Table.

9. In the TEA Results and Sensitivity Analysis, supporting assumed AER starch prices with market references would make the economic conclusions more robust and credible.

Reply: Thank you for your feedback. In the market, AER-starch is not commercially available on the market. Thus, its purchasing cost was estimated based on the price of cassava starch, which can be considered a comparable product. In this context, the price was obtained from a local grocery store. We have added the market reference to the manuscript.

10. Discussing scale-up factors such as mixing, heat transfer, enzyme kinetics, and viscosity would improve the realism of extrapolating laboratory performance to industrial conditions.

Reply: Thank you for your feedback. We have added the following information to the manuscript:

Factors such as mixing and heat transfer, as well as viscosity and enzyme kinetics, should be considered during scale-up. For example, an increase in viscosity can reduce bubble collapse, reducing the cavitation effect and mass transfer rates. Moreover, the distribution of ultrasound energy is influenced by reactor size because cavitation is not uniform across the reactor. Moreover, an increase in temperature requires precise temperature control to preserve enzyme activity [18]. On the other hand, as acoustic cavitation may negatively affect enzyme stability, the ultrasound is commonly applied as a pretreatment rather than simultaneously.

In practice, perfect mixing conditions cannot be achieved at a large scale, and there is no single consensus approach for scale-up [19]. Instead, a scale-up approach could rely on maintaining comparable laboratory-scale operating conditions and assuming they can be extrapolated, while acknowledging unavoidable gradients in temperature, pH, and concentration at the industrial scale. In this approach, we assumed that the HD and SC of the hydrolysates obtained at the laboratory scale would be reproduced at larger scales under the same hydrolysis conditions (e.g., AER-starch concentration, hydrolysis time, temperature, etc.).

11. Expanding the literature review to include recent advances in ultrasound-assisted enzymatic hydrolysis and cavitation mechanisms would enhance the scientific context in the Introduction section.

Reply: Thank you for your feedback. We have added the following information to the manuscript:

Depending on the matrix and enzyme, the ultrasound operation parameters have different effects. For example, during the preparation of hydrolyzed egg yolk powder after ultrasound pretreatment, cavitation and excessive ultrasound led to gradual increases and decreases in surface hydrophobicity and free sulfhydryl groups, as observed with increasing ultrasound time. In consequence, the protein swollen with disulfide bonds becomes more aggregated, and the reconstruction of these bonds was facilitated by cavitation [11]. Cavitation also generates microjets and radicals, thereby increasing selective disruption of the cell wall in lignocellulosic matrices such as brewer’s spent grains. Moreover, certain combinations of ultrasound power and frequency can induce structural reorganization, which restricts cellulose accessibility [9]. For example, in that study, the highest cellulose concentration was observed at 130 kHz and 200 W, whereas the lowest was observed at 5 kHz and 100 W. Another factor with a key role in the cavitation phenomenon is the ultrasound intensity, which influences bubble nucleation, growth, and collapse. Ultrasound intensities ranging from 0.5 to 8 W mL^-1^ were evaluated in the pretreatment enzymatic hydrolysis of polygalacturonic acid [12]. Excessively high intensities increase fluid circulation, leading to reduced cavitation efficiency due to foam formation and excessive air entrainment, particularly at 8 W mL^-1^.

12. Including a brief comparison with existing commercial glucose syrup production routes could further strengthen the TEA by highlighting the competitive position and market potential of glucose syrup derived from AER starch.

Reply: Thank you for your feedback. We have added the following information to the manuscript:

Commercial glucose syrup is commonly produced from corn or cassava starch via enzymatic hydrolysis with α-amylase and glucoamylase, using a well-established industrial route. While the conventional process benefits from economies of scale, only conventional refined starch feedstocks are used. The production of glucose syrup from AER-starch could represent an alternative pathway for valorizing extraction residues and intensify the use of raw materials, contributing to the circular economy. Although the proposed enzymatic hydrolysis process is similar to the conventional process, using AER-starch may offer greater sustainability and reduced waste.

13. Incorporating a short discussion on operational risks, such as feedstock variability or potential equipment downtime, would add practical context and help readers appreciate real-world implementation factors.

Reply: Thank you for your feedback. We have added the following information to the manuscript:

In a real scenario, factors such as AER-starch composition and equipment downtime can pose risks to the process implementation. For example, AER-starch composition (e.g., moisture content, impurities, etc) can affect the efficiency of the hydrolysis. Therefore, at the industrial level, appropriate quality control of raw materials should be established. Moreover, equipment downtime associated with ultrasound pretreatment, the enzymatic reactor vessel, and evaporation could affect plant productivity. Therefore, factors such as preventive maintenance and process monitoring should be considered for process implementation in a real scenario.

Reviewer #2: The reply and manuscript’s text adjustments of Reviewer #2 comments were highlighted in turquoise. We thank the Reviewer for the thorough review of the manuscript.

Section 2.5

Expand/clarify process modeling details. A paragraph can be added to describe the following:

1. Thermodynamic/physical property methods used in SuperPro Designer

Reply: Thank you for the comment. The following paragraph has been added to the text in the "Process simulation model" section:

The process was modeled using the ideal thermodynamic property method, which assumes ideal mixing behavior. Physical properties of compounds such as water, starch, and glucose were obtained from the SuperPro Designer database. New components, such as the hydrolyzed starch fractions, were entered into the software using physical properties reported in the literature.

2. How non-conventional components such as AER-starch and protiens were represented. Pseudo-components or user defined?

Reply: Thank you for the comment. Non-conventional components such as AER-starch and proteins were defined as user-defined components in SuperPro Designer. Their physical and thermodynamic properties (e.g., density, heat capacity, solubility assumptions) were specified based on literature values and process-specific assumptions.

3. Whether full energy balances were solved, how electricity/steam demands were estimated for the evaporation, filtration and mixing steps

Reply: Full mass and energy balances were solved for all unit operations in SuperPro Designer. The software automatically calculated electricity consumption for mixing and pumping based on equipment specifications and operating conditions. Steam requirements for the evaporation step were estimated using the built-in heat duty calculations derived from the specified inlet–outlet temperatures and the thermophysical properties of the process streams. For filtration, SuperPro Designer estimated utility demands (electricity) from the pressure drop and filtration area defined for the equipment. These automated calculations allowed the determination of total utility consumption for each scenario evaluated.

Section 3.3

3. Strengthen the justification for excluding ultrasound in the TEA flowsheet. It was mentioned that ultrasound at high starch concentration does not significantly improve performance and would increase COM; however, in the results it was stated only that ultrasound was not considered further.

Reply: Thank you for your feedback. We have added the following information to the manuscript in the materials and methods section:

The AER-starch ultrasound-assisted pretreatment used an ultrasound unit (P-21/V104), a cluster of four 16 kW ultrasonic processors with temperature control.

- Include a simplified alternative flowsheet with ultrasound units (even if only described qualitatively).

Reply: Thank you for your feedback. We have added the alternative flowsheet as the new Fig 2.

- Provide a short comparative COM estimate (e.g., “preliminary analysis indicated that including ultrasound increased COM by X–Y% at 350 g·L⁻¹ with no significant yield benefit”).

Reply: Thank you for your feedback. We have added the following text:

On the other hand, ultrasound pretreatment did not significantly enhance process performance at higher AER-starch concentrations. Moreover, the higher capital cost of the UAE unit and the additional pretreatment time increase the overall process cost. For example, a preliminary analysis indicated that the COM of the UCEH process was US$3.61 kg^-1^, which is 24% higher than the COM obtained in the CEH process (US$2.90 kg^-1^) when the higher AER-starch concentration was considered. In this context, the techno-economic assessment only considered the CE process for simulation.

- Clearly state why ultrasound is attractive at low concentrations (process intensification) but not at the industrially relevant high concentrations.

Reply: Thank you for your feedback. We have added the following text:

When the ultrasound pretreatment is applied at low AER-starch concentrations, process performance is enhanced by the cavitation effects that improve enzyme accessibility. However, at higher starch concentrations, the increased viscosity attenuates cavitation, reducing the propagation of ultrasonic energy. As a result, ultrasound pretreatment did not yield significant improvements in yield. Moreover, the COM increased due to higher capital and operating costs of the UAE unit, as well as additional energy consumption and pretreatment time. Therefore, under these conditions, ultrasound pretreatment is not economically feasible at industrially high AER- starch concentrations.

Section 3.4

5. Clarify the economic framework more systematically. Right now, elements like discount rate (7%), payback, ROI, IRR, and NPV are described in the results, but not all main cash-flow assumptions are grouped in one place.

I’d suggest to add a small “Economic assumptions” table/box containing:

- Project lifetime (years).

- Discount rate.

- Tax rate (or explicitly state that taxes are neglected).

- Depreciation method and period.

- Working capital assumptions (if any).

- Currency and reference year for all costs (e.g., “all costs in 2024 USD”).

- Basis (330 days/year, 5000 L batch, plant availability, etc.).

That makes the TEA more standard and easier to compare with other studies.

Reply: Thank you for the comment. This information has been compiled and is now presented as supplementary material in S1 Table.
