# Supplementary material for: Enzymatic hydrolysis of starch from the anthocyanin extraction residue (AER-starch) with ultrasound pretreatment: A techno-economic assessment
Source: PLoS One. 2026 Mar 2;21(3):e0343968. doi: 10.1371/journal.pone.0343968 (PMC12952594; doi:10.1371/journal.pone.0343968)
Supplement: Attachment — Submitted filename: Response_to_Reviewers_auresp_2.docx [file pone.0343968.s005.docx]

Review Comments to the Author:

Reviewer #1: The reply and manuscript’s text adjustments of Reviewer #1 comments were highlighted in yellow. We thank the Reviewer for the thorough review of the manuscript.

• Please standardize units/notation (e.g., g/L, kg/kg, US$/kg) and define all acronyms at first mention (AER, SC, HD, CEH/SEH/UCEH) to improve readability.

Reply: Thank you for the comment. We have revised the manuscript to standardize all units and notation across the text, tables, and figures. Additionally, all acronyms and abbreviations were revised.

• Please add a brief justification (or citation) for the selected enzyme dosages and hydrolysis duration to strengthen comparability with prior studies.

Reply: Thank you for the comment. The enzyme dosages and hydrolysis durations were selected based on the technical datasheets and usage recommendations provided by the enzyme supplier (Proenzimas). This information has been included in the Materials and Methods section, together with a reference to the supplier’s technical documentation.

• Please clarify the intended syrup product specification (e.g., target solids content and typical sugar profile) so the selling-price assumption is easier to interpret.

Reply: Thank you for the comment. We assumed a final composition of the glucose syrup as approximately 90% glucose, 1.2% maltose, and 1.2% maltotriose, with traces of minor soluble and proteins. This information was added to the text.

• For transparency, please specify whether costs are reported in constant-year USD and include the price year/inflation basis in Table S1 to support reproducibility.

Reply: Thank you for the comment. We have added the following information to Table S1:

Reference year: 2025 (all costs expressed in constant 2025 USD.

Inflation index: 2025 (the capital cost was adjusted using SuperPro V14 built-in values of the Chemical Engineering Cost Index).

Reviewer #2: (No Response)
